# Interactive effects of severe drought and grazing on the life history cycle of a bioindicator species

**DOI:** 10.1002/ece3.4432

**Published:** 2018-09-01

**Authors:** Sarah Rebecah Fritts, Blake A. Grisham, Robert D. Cox, Clint W. Boal, David A. Haukos, Patricia McDaniel, Christian A. Hagen, Daniel U. Greene

**Affiliations:** ^1^ Department of Biology Texas State University San Marcos Texas; ^2^ Department of Natural Resources Management Texas Tech University Lubbock Texas; ^3^ U.S. Geological Survey Texas Cooperative Fish and Wildlife Research Unit Texas Tech University Lubbock Texas; ^4^ U.S. Geological Survey Kansas Cooperative Fish and Wildlife Research Unit Kansas State University Manhattan Kansas; ^5^ Phalarope Consulting Clovis New Mexico; ^6^ Department of Fisheries and Wildlife Oregon State University Bend Oregon; ^7^ Southern Timberlands Technology Weyerhaeuser Company Columbus Mississippi

**Keywords:** climate change, drought, grazing, lesser prairie‐chicken, sand shinnery oak grasslands, Southern High Plains

## Abstract

We used the lesser prairie‐chicken (*Tympanuchus pallidicinctus*), an iconic grouse species that exhibits a boom–bust life history strategy, on the Southern High Plains, USA, as a bioindicator of main and interactive effects of severe drought and grazing. This region experienced the worst drought on record in 2011. We surveyed lesser prairie‐chicken leks (i.e., communal breeding grounds) across 12 years that represented 7 years before the 2011 drought (predrought) and 4 years during and following the 2011 drought (postdrought). Grazing was annually managed with the objective of achieving ≤50% utilization of aboveground vegetation biomass. We used lek (*n* = 49) count data and covariates of weather and managed grazing to: (a) estimate long‐term lesser prairie‐chicken abundance and compare abundance predrought and postdrought; (b) examine the influence of annual and seasonal drought (modified Palmer drought index), temperature, and precipitation on long‐term lesser prairie‐chicken survival and recruitment; and (c) assess and compare the influence of grazing on lesser prairie‐chicken population predrought and postdrought. Lesser prairie‐chicken abundance was nearly seven times greater predrought than postdrought, and population declines were attributed to decreased survival and recruitment. The number of days with temperature >90th percentile had the greatest effect, particularly on recruitment. The population exhibited a substantial bust during 2011 and 2012 without a boom to recover in four postdrought years. Adaptive grazing positively influenced the population predrought, but had no effects postdrought. Results suggest that the severe drought in 2011 may have been beyond the range of environmental conditions to which lesser prairie‐chickens, and likely other species, have adapted. Land management practices, such as grazing, should remain adaptive to ensure potential negative influences to all species are avoided. Increasing habitat quantity and quality by reducing habitat loss and fragmentation likely will increase resiliency of the ecosystem and individual species.

## 1 INTRODUCTION

Anthropogenic‐induced climate change has altered the frequency and intensity of weather to the extent that these events are affecting biotic systems. Species’ responses to global climate change include shifts in distributions, changes in population sizes, and alterations in reproductive phenology (Charmantier & Gienapp, 2014; Thomas et al., 2004). In arid landscapes, where precipitation is a key limiting resource (Noy‐Meir, 1973; Schwinning & Sala, 2004), precipitation pulses (i.e., discrete, infrequent, and unpredictable precipitation events) are important drivers of ecosystem structure and function (Ehleringer, Schwinning, & Gebauer, 1999; Schwinning & Sala, 2004). Climate change‐induced alterations in the seasonality and variability of precipitation events likely will have cascading effects from soil moisture to plant and wildlife species (Weltzin et al., 2003). In fact, changes in precipitation frequency and magnitude have the potential to transform entire landscapes over the course of the next 50 years.

As species and ecological systems are affected by alterations in climate, their resilience (the ability of a system to return to an equilibrium state after a temporary disturbance) and resistance (a measure of the persistence of systems and their ability to absorb change and disturbance while maintaining the same relationships among populations or state variables (Chambers et al., 2017; Holling, 1973) will influence population persistence. In arid ecosystems, the boom–bust life history strategy is not uncommon (Arthington & Balcombe, 2011; Dickman, Greenville, Beh, Tamayo, & Wardle, 2010; Kingsford, Curtin, & Porter, 1999) and likely evolved in response to pulses in precipitation with increased abundance during wet years (booms) and decreased abundance during dry years (bust). However, species and systems may have low resistance and resilience to future climatic conditions if they are outside of the range to which they have adapted. Therefore, understanding and incorporating resistance and resilience of species and systems are becoming increasingly important to conservation planning and adaptive management strategies, particularly in regard to the management of wildlife and their habitats, to facilitate population persistence in a changing climate (Chambers et al., 2017; Hannah et al., 2002; Lawler, 2009; Mawdsley, O'Malley, & Ojima, 2009; Scheffer, Carpenter, Foley, Folke, & Walker, 2001).

In addition to ongoing effects of climate change, shifts in the primary ecological drivers that historically maintained landscapes have affected the structuring of plant and animal communities. A loss of ecological drivers is especially true for grasslands, which represent one of the most altered ecosystems in North America (Samson & Knopf, 1994). Within the Southern High Plains, a semiarid subregion at the southwestern extent of the Great Plains, traditional ecological drivers of drought, fire, and grazing have often changed as native prairies have been converted to other land use types (Milchunas, Lauenroth, Chapman, & Kazempour, 1989; Milchunas, Sala, & Lauenroth, 1988; Samson & Knopf, 1994, 1996; Savage, 1937). Combined, climate change‐driven alterations in temperature and extreme drought occurrence and alterations in other ecological drivers may influence wildlife on the Southern High Plains directly and through changes in vegetation composition and structure (reviewed in Grisham, Godar, Boal, & Haukos, 2016).

Understanding the roles of ecological drivers on ecosystem structure and function is a vital issue in conservation (Knopf & Samson, 1997), particularly in grassland ecosystems where biota are adapted to the periodic and intermittent effects of drivers. One important ecological driver in grassland structure and function is grazer population densities and species (McNaughton, Ruess, & Seagle, 1988). In history, several species, including American bison (*Bison bison*), pronghorn (*Antilocapra americana*), elk (*Cervus canadensis*), and mule deer (*Odocoileus hemionus*), grazed the Southern High Plains (Peterson & Boyd, 1998); however, humans have influenced grazing patterns by replacing nomadic American bison and elk with domestic cattle, which have different foraging ecologies (Plumb & Dodd, 1993). These changes in grazing regimes can act independently or with other drivers to alter the vegetation structure and function over vast landscapes, thereby affecting wildlife populations.

Species that are range‐restricted, isolated, and at the periphery of their range may be at greater risk of extinction or extirpation than those with wide distributions (Gibson, Van der Marel, & Starzomski, 2009; Thomas et al., 2004). As such, the sensitivity of species at the edge of their range makes them ideal bioindicators to assess the influences of climate change and other alterations occurring within their habitats. The lesser prairie‐chicken (*Tympanuchus pallidicinctus*), an iconic grouse species of the Great Plains, is declining, in part because of habitat alterations and changes in disturbance regimes that have historically maintained the species’ habitat (Bailey & Painter, 1994; Dhillion, McGinley, Friese, & Zak, 1994; Grisham et al., 2013; Hagen & Giesen, 2005; Hagen, Jamison, Giesen, & Riley, 2004). Within Sand Shinnery Oak Prairies on the Southern High Plains, which represents both the southernmost and westernmost extent of the lesser prairie‐chicken range, populations exhibit a boom–bust reproductive strategy, a life history adaptation where adult females invest more in their own survival when weather conditions are suboptimal and allocate reproductive efforts during relatively cool, wet breeding seasons (Grisham et al., 2013; Patten, Wolfe, Shochat, & Sherrod, 2005b). Although lesser prairie‐chickens evolved with recurring drought within the Southern High Plains, increases in drought magnitude and frequency may hinder population recovery during nondrought years (Chen & Newman, 1998; Peterson & Boyd, 1998; Wester et al., 2007) due to mismatch between phenology of rainfall events, life span, and reproductive efforts.

Landscapes within the Southern High Plains and their species composition have been drastically reshaped by interannual variability in precipitation and recurring drought, which, historically, occurs at 5‐ to 10‐year intervals (Chen & Newman, 1998; Peterson & Boyd, 1998; Wester et al., 2007). Hence, drought is one of the main historical drivers of plant and wildlife populations on the Southern High Plains (Grisham, Godar & Griffin, 2016; Haukos, 2011; Samson, Knopf, & Ostlie, 2004). However, multiscenario climate models identify the Southern High Plains as a hot spot of extreme impacts of climate change (Diffenbaugh, Giorgi, & Pal, 2008). Extreme impacts include decreases in precipitation and increases in temperature, drought frequency, and drought intensity (Cook, Ault, & Smerdon, 2015; Karl et al., 2010; Oliver, Brereton, & Roy, 2013; Peterson, Stott, & Herring, 2012). The number of days >37.8°C is expected to quadruple to approximately 26–28 days under high emission scenarios by mid‐century, and summers in the region are expected to have less rainfall and longer periods without rainfall (Shafer et al., 2014). The region experienced the worst drought on record in 2011 with >100 days >37.8°C and set new records for the hottest summer since documentation began in 1895 (Nielsen‐Gammon, 2012). Cumulative rainfall during the first 10 months of 2011 was nearly six times lower than the 30‐year average of 43.94 cm at the West Texas Mesonet Site (Lubbock, TX, USA). Substantial increases in year‐to‐year variability in rainfall patterns and more severe storms also are expected (Christian, Christian, & Basara, 2015; Cook et al., 2015).

Synergistically, drought and grazing can alter vegetation communities differently than each of the drivers independently, which may magnify their combined influence on wildlife populations (Loeser, Sisk, & Crews, 2007). Moreover, humans have exacerbated the rate and intensity of drought impacts by influencing the process of desertification through unmanaged grazing (Grover & Musick, 1990). Locally, grazing can shift the structure and composition of vegetation and result in woody shrub encroachment (e.g., Grisham, Borsdorf, Boal, & Boydston, 2014; Haukos & Smith, 1989; Peterson & Boyd, 1998; Riley, Davis, Ortiz, & Wisdom, 1992). Vegetation transitions occur in part because grazing intensity directly affects the time needed for vegetation to recover postdrought, which can take ≥20 years if overgrazed during a drought, and likely is exacerbated in semiarid or precipitation‐limited ecosystems (Albertson, Tomanek, & Riegel, 1957). Although the effects of drought (Grisham et al., 2013; Merchant, 1982; Peterson & Silvy, 1994; Ross, Haukos, Hagen, & Pitman, 2016a) and grazing (Fritts et al., 2016; Jackson & DeArment, 1963; Silvy, Peterson, & Lopez, 2004) on prairie grouse have been surmised, there are considerable knowledge gaps regarding potential interactive effects of these two important ecological drivers, particularly at the core habitat complex scale (i.e., ≤10,200 ha) (Bidwell et al., 2003).

To assess the main and interactive effects of drought and grazing on lesser prairie‐chicken populations, we used grazing data from 7 years before the 2011 drought “predrought” (2004–2010) and 5 years during and after the 2011 drought “postdrought” (2011–2015). In this study, grazing was adaptively managed annually based on vegetation biomass; therefore, we examined lesser prairie‐chicken response to alterations in grazing pressure because of drought‐induced decreases in vegetation biomass. In particular, we had three objectives: (a) to estimate long‐term lesser prairie‐chicken abundance and compare abundance predrought and postdrought; (b) to examine the influence of annual and seasonal drought (modified Palmer drought index), temperature, and precipitation on long‐term lesser prairie‐chicken survival and recruitment; and (c) to assess and compare the influence of grazing on lesser prairie‐chicken population predrought and postdrought. Lesser prairie‐chicken abundance declines following severe drought (Jackson & DeArment, 1963; Ross et al., 2016a; Sullivan, Hughes, & Lionberger, 2001), and we hypothesized abundance estimates would severely decline following the 2011 drought due to declines in both survival and recruitment (Grisham et al., 2013, 2014; Patten, Wolfe, Shochat, & Sherrod, 2005a; Patten et al., 2005b). Because appropriate grazing pressure can stimulate biomass production that can be used for nesting and thermoregulatory cover, we hypothesized that lesser prairie‐chicken abundance would be positively correlated with grazing predrought (Fritts et al., 2016; Hagen, Grisham, Boal, & Haukos, 2013). At last, because survival is dependent on microhabitat and microclimate availability potentially because temperature and wind affect metabolic rates (Patten et al., 2005a; Sherfy & Pekins, 1995), we hypothesized that lesser prairie‐chicken abundance, survival, and recruitment would be negatively related to grazing during/postdrought.

## 2 MATERIALS AND METHODS

### 2.1 Study site

Milnesand Prairie Preserve (hereafter “Preserve,” 33 69°N, 103 38°W) was a 75‐km^2^_,_ privately owned rangeland in Roosevelt County, New Mexico, USA, on the western edge of the Southern High Plains. The preserve was primarily used for livestock production and management of lesser prairie‐chicken habitat. Strahan (2008) provides a floristic survey of Milnesand Prairie Preserve.

The conservation strategy of Milnesand Prairie Preserve was to maintain and improve habitat for lesser prairie‐chickens through adaptive grazing management, while maintaining the producer's financial viability. The grazing rotation and animal unit months had a goal of 50% utilization of available forage (actual utilization across pastures and years of study (mean ± *SD*) was 51% ± 23%). Although considerations of stocking rate depend largely on precipitation, moderate rates often are aimed at achieving 35%–50% utilization (Benson, Zhu, Farmer, & Villalobos, 2011; Holechek, Thomas, Molinar, & Galt, 1999). During this study, the grazing system was a cow–calf operation split into two herds. Calving occurred in January, with the annual calf‐crop sold between October and November. Pastures were 731 ± 250 ha (mean ± *SD*) in size and were grazed throughout the duration of the study, and herds were rotated through most pastures at least once each year with periods of rest with the goal of achieving a 50% utilization rate (Table 1). In addition, cattle were removed from some pastures from late April–mid‐May because tannins in sand shinnery oak (*Quercus havardii*) catkins can cause decreased recruitment in cattle if ingested in large quantities (Bausch & Carson, 1981). We calculated the number of cow days/ha as the number of cattle in the pasture multiplied by the number of days the cows were in a specific pasture divided by the pasture size (ha). Cow days/ha and percent utilization (Table 1) were not related (Pearson's R correlation coefficient = −0.17).

**Table 1:** The number of birds counted on a lek (mean ± SD) per observation, the maximum number of birds seen on a lek, the number of cow days/ha (cow days), percent utilization (% Util), annual precipitation (cm) average annual modified Palmer drought index (Avg PMDI), minimum PMDI (min PMDI), annual maximum temperature (°C; max temp), and the number of hot days (days above the 90th percentile) in pastures containing 49 lesser prairie‐chicken (*Tympanuchus pallidicinctus*) leks in Milnesand Prairie Preserve, New Mexico, 2004–2015. Blank spaces indicate missing data. Mean ± *SD* are reported when values differed by pasture

Covariate	Predrought							Postdrought				
	2004	2005	2006	2007	2008	2009	2010	2011	2012	2013	2014	2015
Avg birds	8 ± 9	11 ± 9	15 ± 13	8 ± 8	9 ± 12	5 ± 7	3 ± 3	6 ± 10	3 ± 5	2 ± 4	1 ± 2	1 ± 2
Max birds	35	36	67	44	65	28	10	51	14	21	5	9
Cow days		2.0 ± 0.8	1.0 ± 0.5	1.2 ± 0.5	0.9 ± 0.6	1.4 ± 0.9	1.0 ± 0.7	0.4 ± 0.3		0.2 ± 0.1	0.2 ± 0.2	0.3 ± 0.2
% Util				61 ± 24	50 ± 27	46 ± 18	52 ± 27		59 ± 23			
Precipitation	69.55	33.7	47.78	45.57	31.88	43.1	43.36	20.47	21.11	46.46	31.678	66.09
Avg PMDI	0.53	3.36	−1.55	0.09	−1.36	−2.22	0.85	−3.74	−4.56	−3.25	−2.62	2.72
Min PMDI	−3.85	−0.06	−3.92	−2.59	−3.31	−3.55	−1.09	−6.18	−5.86	−5.01	−3.4	−0.09
Max temp	38.33	38.89	39.44	40.56	40.00	39.44	39.44	42.78	41.11	40.00	41.11	40.56
Hot days	27	22	41	12	30	37	36	84	59	35	27	44

Weather data were collected approximately 60 km from the study site (Portales, New Mexico (latitude = 34.17417, longitude = −103.352). From 1932 to 2015, the annual precipitation was (mean ± *SD*) 40.5 ± 13.7 cm (National Oceanic and Atmospheric Administration [NOAA], 2017a,b). The majority of precipitation occurs in July and August on our study site (NOAA, 2017a,b). The 25th percentile of annual precipitation from 1950 to 2015 was 32 cm and 75th percentile was 46 cm (NOAA, 2017a,b). Although 2011 received only slightly less rain than 2012, all but 1.3% occurred after the breeding/brood rearing season (during/after June). Maximum annual temperature was 1.7°C hotter in 2011 than any other year during the study. From 2000 to 2014, the 90th percentile temperature of daily maximum temperature was 35°C. The number of days with a temperature >90th percentile was nearly 1.5 times greater in 2011 than any other year of study.

### 2.2 Lesser prairie‐chicken surveys

We counted lesser prairie‐chickens on known active leks (*n *=* *49) from March 17 to April 27 in 2004–2015. We defined leks as an area having ≥3 actively displaying males. To find leks, we conducted roadside surveys throughout the study site. We stopped every 0.6 km to perform visual and auditory searches for lesser prairie‐chickens. Once we located a lek, we returned to the lek 1–5 times per season to flush lekking individuals to get an accurate count. We surveyed from 30 min before sunrise to 4 hrs after sunrise.

### 2.3 Influence of Annual Weather and Grazing on Demographic rates

We estimated abundance, apparent survival (deaths and emigrations), and recruitment (births and immigrations) and assessed the relationships between both weather and grazing covariates and survival and recruitment using multiseason open N‐mixture models with function pcountOpen in package unmarked (Fiske & Chandler, 2011) in R version 3.0.3 (R Core Team, 2014). N‐mixture models can provide robust population trends for lekking species when data are sparse (McCaffery, Nowak, & Lukacs, 2016). Open population N‐mixture models fit the model of Dail and Madsen (2011), which is a generalized form of the Royle (2004) N‐mixture model. These models were specifically designed to use count data from unmarked individuals to estimate: *γ*, the recruitment rate (births and immigration, the finite rate of increase, or the maximum instantaneous rate of increase); and *ω*, the apparent survival rate (deaths and emigrations; Fiske et al., 2017).

For all N‐mixture models, we followed a four‐step process. First, we determined the appropriate distribution of the response variable (count data) by comparing null models with zero‐inflated Poisson, Poisson, and negative binomial distributions with AIC (Akaike, 1998). Second, we identified significant predictors of detection by assessing the global detection model with daily rainfall, daily maximum temperature, and effort (the number of times a lek was visited) on the detection process. We omitted variables that had a beta value with a 95% confidence interval that overlapped 0. In the same way, we examined whether the number of individuals counted on each lek the year prior was a predictor of initial abundance. At last, we included covariates of grazing or weather on the survival or recruitment process to assess their influence on lesser prairie‐chicken populations. For recruitment models, we used autoregressive population dynamics, which models recruitment as gamma*N[i, t‐1]. For survival models, we used constant population dynamics, which indicates no relationship between survival and recruitment. Both autoregressive and constant population dynamics use a Markovian process to model the latent abundance state following the initial sampling period in which survivors are modeled as *S*_*it*_ *~ Binomial*(*N*_*it‐1*_ *, w*_*it*_) and recruits follow *G*_*it*_ *~ Poisson*(γ_*it*_).

To assess the influence of annual weather on lesser prairie‐chicken populations, we combined 12 years of lesser prairie‐chicken lek counts. We included 10 variables; annual precipitation (NOAA 2017a), annual average of the modified Palmer drought index (PMDI; NOAA 2017b), and the annual minimum PMDI, annual maximum temperature, and annual number of hot days (the number of days over the 90th percentile in temperature; Table 1) on either the survival or recruitment process. In addition, we included the same five variables with a 1‐year time lag in separate models. We used one of the 10 weather covariates (each of the five variables measured during the same year as the lesser prairie‐chicken population estimates and each with a 1‐year time lag) as a continuous covariate on either recruitment or survival for a total of 20 weather models. We modeled only one covariate on either survival or recruitment because pcountOpen is particularly limited (e.g., slow and has difficulty converging) when including covariates on gamma or omega (Fiske et al., 2017). We estimated annual abundance using the AIC best model and compared between predrought and during/postdrought using an analysis of variance. Using the same methods as above, we assessed the influence of seasonal weather on lesser prairie‐chicken demographics. We used the same response variable (count data from 2004 to 2015) and seasonal (winter: December– February, breeding: March–May, summer: June–August, and fall: September–November) total rainfall and average PMDI during the same year and with a 1‐year time lag as continuous independent variables. We did not assess maximum temperature, minimum PMDI, or hot days seasonally because nearly all were in the summer; thus, those were assessed using the annual covariates. We also did not assess weather influences from the winter or spring the year before because we assumed the weather from >9 months before did not affect the population.

To assess the influence of grazing predrought and postdrought, we analyzed data similar to assessing weather covariates, except we divided data into two time periods: predrought (2004–2010) and during/postdrought (2011–2015). We separated these time periods because we hypothesized that the influence of grazing would be different predrought compared to postdrought. We used the number of cow days/ha as a continuous explanatory covariate on either the recruitment or survival process. Land managers did not record the number of cows in the herd in 2012; therefore, this year was omitted from the grazing assessment. In addition, utilization was recorded intermittently and was not used as a covariate.

## 3 RESULTS

**Figure 1:**
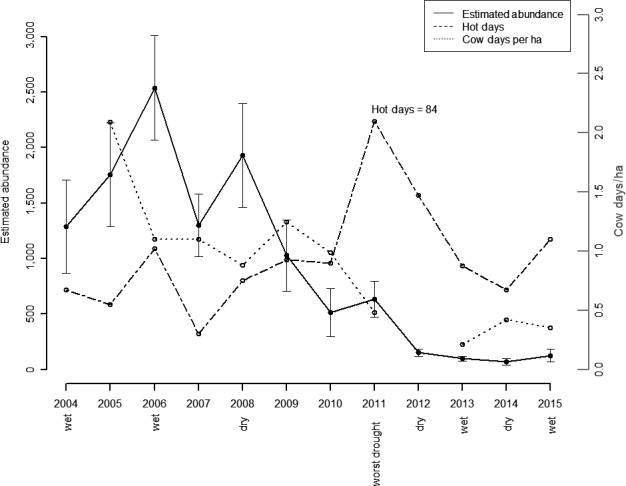
Average estimated abundance of lesser prairie‐chickens (*Tympanuchus pallidicinctus*) in 49 leks, the number of cow days/ha in pastures containing leks, and annual precipitation in Sand Shinnery Oak Prairie Ecoregion of eastern New Mexico. Wet years are above the 75th percentile of annual precipitation totals since 1950, whereas dry years are below the 25th percentile of annual precipitation totals since 1950. Bars represent 95% confidence intervals on abundance estimates

**Figure 2:**
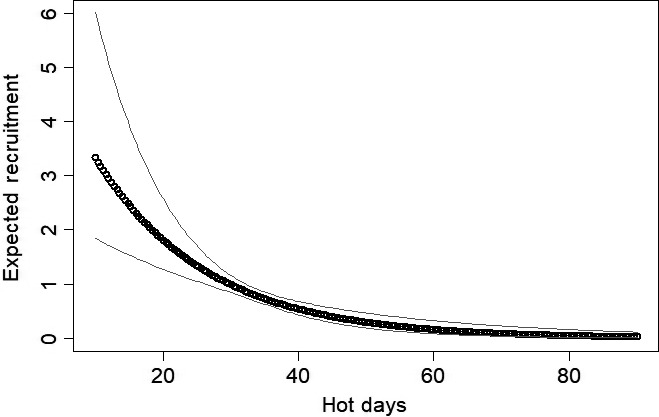
The number of annual days with a maximum temperature >90th percentile (hot days) negatively influenced lesser prairie‐chicken (*Tympanuchus pallidicinctus*;* n* = 49 leks) recruitment in Milnesand Prairie Preserve, New Mexico, from 2004 to 2015. Gray lines represent 95% confidence intervals

**Figure 3:**
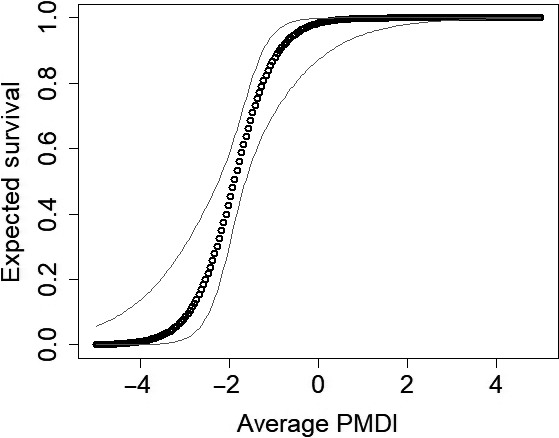
Average annual Palmer’Modified Drought Index (PMDI) positively influenced lesser prairie‐chicken (*Tympanuchus pallidicinctus*;* n* = 49 leks) survival in Milnesand Prairie Preserve, New Mexico, from 2004 to 2015. Gray lines represent 95% confidence intervals

**Figure 4:**
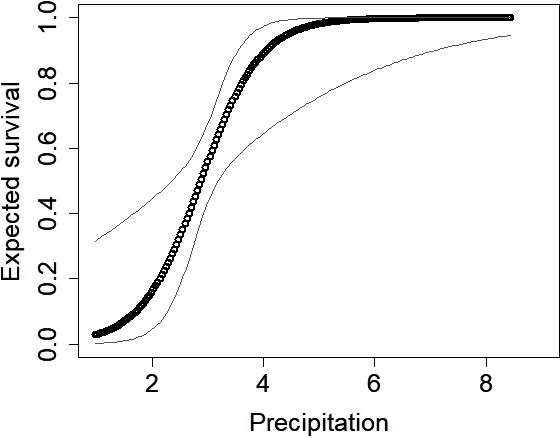
Spring precipitation (cm) positively influenced lesser prairie‐chicken (*Tympanuchus pallidicinctus*;* n* = 49 leks) survival in Milnesand Prairie Preserve, New Mexico, from 2004 to 2015. Gray lines represent 95% confidence intervals

Lesser prairie‐chicken abundance estimates were nearly seven times greater predrought than postdrought (*F*_1,10_ = 16.46; *p* < 0.01) and failed to rebound following the 2011 drought (Figure 1). Decreases in abundance can be attributed to reductions in mostly recruitment, but survival may have decreased as well. Hot days on the recruitment process was the AIC best model and accounted for 100% of model cumulative weight (Table 2). Recruitment significantly decreased once the number of hot days was above 20 days (log‐scale β = −1.14, *SE* = 0.24, 95% CI = −1.61 to −0.67; Figure 2). The AIC best model of survival included average PMDI and accounted for 67% of weights of models that included survival (Table 2). As PMDI increased to above −2, survival increased rapidly (Figure 3). Spring precipitation accounted for 32% of the survival model weights (Table 2). Survival was approximately 50% when spring precipitation was approximately 3 cm and maximized when spring precipitation was slightly over 4 cm (Figure 4).

**Table 2:** Open population N‐mixture models to assess the influence of annual precipitation, annual average of modified Palmer drought index (PMDI), annual minimum PMDI, seasonal precipitation, and seasonal average PMDI during the same year each with a 1‐year time lag on lesser prairie‐chicken (*Tympanuchus pallidicinctus*) survival and recruitment over 49 leks in Milnesand Prairie Preserve, New Mexico, USA, in 2005–2015. Model selection based on Akiake's information criteria (AIC) model fit, number of parameters (*K*), the difference AICc from the best fit model (ΔAICc), and model weights (*w*_i_). Beta values of covariates and 95% confidence intervals are listed

Demographic	Covariate	Time period	ΔAICc	*w*_i_	β	LCL	UCL
Recruitment	Hot days	Same year	0.00	1.00	1.14	−1.61	−0.67
Recruitment	Rainfall spring	Same year	0.00	0.97	0.41	0.23	0.60
Survival	Average PMDI	Same year	17.77	1.00	5.41	2.42	8.39
Survival	Hot days	Same year	19.34	1.00	−0.13	−0.21	−0.05
Survival	Average PMDI summer	Same year	9.24	0.98	4.12	1.08	7.16
Recruitment	Average PMDI winter	Same year	10.02	0.99	1.22	0.39	2.05
Recruitment	Precipitation	Same year	22.35	1.00	0.75	0.19	1.03
Survival	Rainfall spring	Same year	10.67	0.99	5.03	1.33	8.72
Recruitment	Average PMDI	Same year	22.77	1.00	0.75	0.20	1.30
Recruitment	Average PMDI summer	Same year	11.05	1.00	0.53	0.14	0.93
Recruitment	Rainfall fall	Same year	13.86	1.00	−1.10	−2.93	0.74
Recruitment	Precipitation	Year before	25.85	1.00	0.61	−0.03	1.25
Survival	Precipitation	Same year	26.77	1.00	2.78	1.17	4.39
Recruitment	Average PMDI fall	Same year	15.88	1.00	0.34	−0.08	0.78
Recruitment	Average PMDI fall	Year before	16.61	1.00	0.21	−0.08	0.50
Recruitment	Maximum temperature	Same year	28.77	1.00	−0.36	−0.58	0.13
Recruitment	Rainfall summer	Same year	17.47	1.00	−0.16	−0.47	0.15
Recruitment	Rainfall summer	Year before	17.47	1.00	0.16	−0.16	0.49
Recruitment	Rainfall fall	Year before	17.73	1.00	0.36	−0.45	1.62
Recruitment	Rainfall winter	Same year	29.91	1.00	−0.16	−0.47	0.15
Recruitment	Maximum temperature	Year before	30.08	1.00	−0.40	−0.51	0.28
Recruitment	Average PMDI	Year before	30.10	1.00	0.07	−0.17	0.31
Recruitment	Minimum PMDI	Same year	30.11	1.00	0.06	−0.15	0.27
Recruitment	Minimum PMDI	Year before	30.15	1.00	0.06	−0.13	0.25
Survival	Average PMDI winter	Same year	18.40	1.00	6.40	0.86	11.94
Recruitment	Average PMDI summer	Year before	18.56	1.00	−0.01	−0.28	0.26
Survival	Minimum PMDI	Same year	32.39	1.00	9.42	4.24	14.61
Survival	Average PMDI	Year before	32.39	1.00	10.53	4.74	16.32
Survival	Minimum PMDI	Year before	32.39	1.00	8.26	3.72	12.80
Survival	Average PMDI fall	Same year	23.88	1.00	2.58	0.64	4.51
Survival	Maximum temperature	Same year	36.96	1.00	−1.40	−2.77	0.70
Survival	Average PMDI spring	Same year	28.36	1.00	6.34	1.23	11.46
Survival	Hot days	Year before	44.21	1.00	−0.08	−0.13	−0.02
Survival	Maximum temperature	Year before	44.76	1.00	−1.24	−2.07	−0.41
Survival	Precipitation	Year before	46.66	1.00	1.98	0.65	3.31
Survival	Rainfall summer	Year before	37.67	1.00	0.70	0.03	1.37
Survival	Average PMDI summer	Year before	39.15	1.00	0.69	−0.47	1.86
Survival	Rainfall fall	Year before	41.40	1.00	0.59	−0.82	2.00
Survival	Rainfall fall	Same year	41.50	1.00	−0.59	−2.12	0.94
Survival	Rainfall winter	Same year	41.57	1.00	0.20	−0.37	0.78
Survival	Rainfall summer	Same year	41.57	1.00	0.20	−0.37	0.78

**Figure 5:**
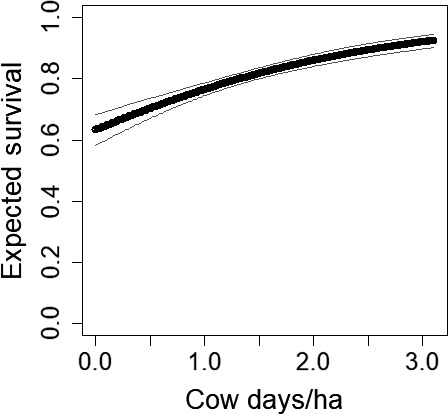
The number of cow days/ha positively influenced lesser prairie‐chicken (*Tympanuchus pallidicinctus*;* n* = 49 leks) survival predrought in Milnesand Prairie Preserve, New Mexico, from 2004 to 2015. Gray lines represent 95% confidence intervals

**Figure 6:**
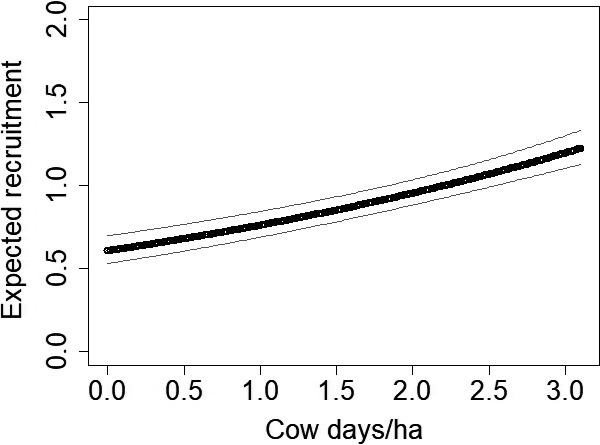
The number of cow days/ha positively influenced lesser prairie‐chicken (*Tympanuchus pallidicinctus*;* n* = 49 leks) recruitment predrought in Milnesand Prairie Preserve, New Mexico, from 2004 to 2015. Gray lines represent 95% confidence intervals

Grazing had positive influences on lesser prairie‐chicken survival (cow days/ha logit‐scale β = 0.54, *SE* = 0.07, *z* = 8.16, 95% CI = 0.41–0.67; Figure 5) and recruitment predrought (cow days/ha log‐scale β = 0.19, *SE* = 0.02, *z* = 9.41, 95% CI = 0.15–0.23; Figure 6), but no influence postdrought. As the number of cow days/ha increased by 1 predrought, both survival and recruitment increased by approximately 0.8 (*SE* = 0.01 and 0.04, respectively).

## 4 DISCUSSION

Using the lesser prairie‐chicken population on the Southern High Plains as a bioindicator of wildlife response to intense drought supports burgeoning evidence that wildlife is negatively affected by above average frequencies of drought and, over the long‐term, may not be adapted to the magnitude of drought expected with climate change. In fact, negative effects of increases in drought magnitude and frequency have been documented for all taxa, including amphibians (Mac Nally, Horrocks, & Lada, 2017), reptiles (Westphal, Stewart, Tennant, Butterfield, & Sinervo, 2016), fish (Jaeger, Olden, & Pelland, 2014), mammals (Ahlers et al., 2015), and birds (Selwood et al., 2015). Like other wildlife species in arid and semiarid grasslands, lesser prairie‐chickens on the Southern High Plains have adaptations that increase resilience to extreme environments and fluctuating weather patterns; however, environmental conditions expected from climate change may be outside of their adaptive potential, particularly in the time frame weather changes are expected to occur. It was apparent from 12‐year population models that lesser prairie‐chicken populations in this ecosystem exhibited a boom–bust life history strategy in which abundance was linked to drought conditions. Typically following a less intense drought followed by a bust period, such as that experienced in 2008, the population rebounds. However, if another drought occurs at a quicker return interval or that is greater in magnitude, populations may not boom before the subsequent bust. Our estimates indicated that the population failed to rebound for at least 4 years following the 2011 drought, suggesting the extreme environmental conditions during 2011 may have been beyond that to which the lesser prairie‐chicken is adapted and/or that the return interval following the 2008/2009 dry period and ensuing low population numbers in 2010 was too short for the population to recover enough to be resilient to the 2011 drought. This bust in population abundance with no subsequent boom has been documented in other bird species as well and indicates low resilience and stability to intensified drought conditions (Selwood et al., 2015).

Declines in lesser prairie‐chicken abundance during periods of drought have been documented throughout the species’ range (Giesen, 1998; Grisham et al., 2013; Merchant, 1982; Rodgers, 2016; Ross, Haukos, Hagen, & Pitman, 2016b). The decreases in abundance observed in our study resulted from both low adult survival and reduced reproductive output, which reduced lesser prairie‐chicken numbers on the Southern High Plains. The number of hot days had the greatest effect on recruitment, which corroborates previous studies that recorded reduced recruitment rates during drought years (Giesen, 1998; Grisham et al., 2014; Merchant, 1982; Ross et al., 2016a). Grisham et al. (2013) suggested aboveaverage winter temperatures, which often are correlated to La Niña events such as in 2011, negatively influence reproductive output, and may lead to nest survival below levels viable for population persistence. Although several mechanisms can result in reduced reproductive output, we surmise that decreased nesting effort and increased nest abandonment ultimately led to fewer broods produced during our study (Grisham et al., 2014; Merchant, 1982). Whether eggs die before or after nest abandonment is unknown, but environmental thresholds of temperature and vapor pressure exist for nest survival (Grisham, Godar, Boal, et al., 2016). Nest success and chick survival greatly influence population growth (Hagen, Sandercock, Pitman, Robel, & Applegate, 2009), both vital to near‐term population persistence. In Sand Shinnery Oak Prairies in Texas, 12 of 15 (80%) radiotagged hens failed to incubate eggs when conditions were similar (e.g., same year and habitat type, but different state) to those in our study (Grisham et al., 2014). Moreover, several lesser prairie‐chicken nest abandonments occurred in 2009 when ambient temperatures exceeded 38°C between four and seven consecutive days (Grisham, 2012). However, precipitation in the 2009 late summer may have increased survival so the effects to the population were not as long‐term as in 2011. Fields, White, Gilgert, and Rodgers (2006) found lower brood survival in Kansas when temperatures exceeded 35º C compared to cooler temperatures, a phenomenon also seen with other grouse species. For example, when operative temperatures were >35°C, nest survival diminished for greater prairie‐chickens (*Tympanuchus cupido*) in Oklahoma (Hovick, Elmore, Allred, Fuhlendorf, & Dahlgren, 2014). Therefore, we suspect thermal stress on incubating hens, eggs, and chicks was responsible for reduced recruitment during the drought in this study.

Land management practices, including grazing, can offset adverse effects of climate change (Greenwood, Mossman, Suggitt, Curtis, & Maclean, 2016; Mawdsley et al., 2009; Pyke & Marty, 2005), particularly if management remains adaptive. In our study, although the directly measured benefits of grazing diminished following the 2011 drought, grazing at substantially reduced rates during and following the drought did not negatively affect lesser prairie‐chicken populations. It is likely that a quadratic relationship between grass canopy cover and demographics exists, as has been found between VOR and nest bowl, nest site selection, and brood locations (Lautenbach 2015), suggesting a tradeoff between cover, movement ability, and escape from predators (Bergerud and Gratson 1988, Hagen, Pitman, Sandercock, Robel, & Applegate, 2007). Tall, thick vegetation can hinder lesser prairie‐chicken ability to move broods through the landscape and reduce their ability to detect predators (Hagen et al., 2007), and grazing can be used to decrease vegetation, particularly in wet years. However, bare ground >35% may be detrimental to nesting hens (Fritts et al., 2016); thus, increases in grazing pressure during or following extreme drought (i.e., comparable to 2011 drought event) may negatively affect survival and recruitment. Measurements of grazing such as utilization and the number of cow days/ha have the potential to provide inference that is prescriptive for land owners and managers on the influence of grazing on wildlife populations.

The timing and magnitude of precipitation events affect plant and wildlife populations (Dale et al., 2001; Fravolini et al., 2005; Heffelfinger, Guthery, Olding, Cochran, & McMullen, 1999; Lusk, Guthery, & DeMaso, 2001; Robertson, Bell, Zak, & Tissue, 2010). Wetter conditions often are optimal for lesser prairie‐chickens on the Southern High Plains, but the importance of precipitation is complex. The timing of precipitation can negatively impact nest and brood survival in the spring, especially when paired with low temperatures (Fields et al., 2006). In addition, the monthly or annual amount of precipitation can be a misleading indicator for lesser prairie‐chicken population resilience and recovery if the magnitude and frequency of precipitation events are ignored. For example, drought conditions, although to a lesser extreme than in 2011, occurred in 2005 and population numbers decreased; however, populations rebounded in 2006 as precipitation increased, particularly during the nesting season (March, 2.59 cm across seven precipitation events, and April, 1.50 cm across four precipitation events, had aboveaverage rain for the month) and end of the summer. Although 2013 was a relatively wet year, 40% of the rain occurred in August and September and may not have led to adequate nesting and brood rearing habitat in March (0.15 cm across two precipitation events) or April (0.05 cm across one precipitation event). March (0.71 cm across four precipitation events) and April 2014 (1.32 cm across three precipitation events) experienced belowaverage precipitation as well.

Climate change and habitat fragmentation interact to affect wildlife populations and communities (Christie, Jensen, Schmidt, & Boyce, 2015; Opdam & Wascher, 2004). Because of large variation in annual habitat conditions, broad landscapes are necessary to provide selection based on habitat quality for wildlife during years of poor primary productivity. Lesser prairie‐chickens are a landscape‐scale species requiring several thousand hectares to fulfill the life history needs of a population (Haukos & Zavaleta, 2016). Lesser prairie‐chickens on the Southern High Plains occupy the Sand Shinnery Oak Prairie ecosystem, which has been considered threatened in New Mexico (Bailey & Painter, 1994) and Texas (Dhillion et al., 1994) for several decades.

In addition to the dry/drought effects experienced in our study following 2011 when the population decline became pronounced, habitat loss and fragmentation in Sand Shinnery Oak Prairies may have exacerbated population declines and contributed to declines prior to the 2011 drought. Although belowoptimal conditions, such as drought, led to the “bust” in the boom–bust population cycle (Hagen et al., 2009), past populations likely had greater potential to recover because of increased habitat connectivity and dispersal ability and greater initial metapopulation numbers to bolster the populations that had been negatively affected (Davis, Horton, Odell, Rodgers, & Whitlaw, 2008; Grisham, 2012). Thus, the low resilience and resistance of lesser prairie‐chicken populations to recent drought likely is exacerbated by habitat loss and fragmentation (Davis et al., 2008; Grisham, 2012; Oliver et al., 2013). Over the long term, intensive, adaptive habitat management will be vital to population persistence.

The resilience and resistance of species and ecosystems to changing environmental conditions depend on several factors. More intact ecosystems likely can better withstand increases in stochastic events (Oliver et al., 2013) as larger landscapes provide broader ranges of microclimates and resources (Hodgson, Moilanen, Wintle, & Thomas, 2011; Oliver, Roy, Hill, Brereton, & Thomas, 2010). Landscapes and terrain that provide microclimates can limit exposure of individuals to extreme environmental conditions and help regulate body temperature and water loss (Dobrowski, 2011; Williams, Shoo, Isaac, Hoffmann, & Langham, 2008). Increased habitat connectivity facilitates an individual's movement across a landscape, as mentioned above; thus, more mobile species may benefit inequitably. If a species is not as mobile, it may, through evolutionary time, alter fitness‐related traits by plastic change or genetic adaptation (Chevin, Lande, & Mace, 2010; Moritz & Agudo, 2013). Lesser prairie‐chickens can have home ranges of thousands of hectares, but they exhibit high site fidelity (Giesen 1994, Riley et al. 1994). The population in our study system not only experiences warmer and drier conditions compared to other populations due to it being the most extreme south and west population, but also the most extreme microclimates, particularly during incubation (Grisham, Godar, & Griffin, 2016; Grisham, Godar, Boal, et al., 2016; Grisham, Zavaleta, et al., 2016). Lesser prairie‐chicken resilience and stability may be increased by efforts to restore marginal croplands back to prairie habitats and to improve habitat quality and connectivity of populations (Ross et al., 2016a), thereby allowing increased immigration and dispersal abilities.

As climate change leads to more variability in rainfall patterns (Christian et al., 2015; Cook et al., 2015), biotic systems will continue to be affected. System responses to climate change are complex and likely nonlinear; therefore, using suitable species as bioindicators will be important for predicting the influences of global change and assessing the role of land management in conservation. For lesser prairie‐chickens, adaptive habitat conservation and management are vital for population persistence beyond 2050 given projections of increased drought frequency and intensity (Christian et al., 2015; Cook et al., 2015; Grisham, Zavaleta, et al., 2016), which, based on results, may further disrupt the boom–bust life history strategy by either reducing the population to levels that cannot rebound quickly or not allowing enough time following an extreme drought to rebound to levels that will be resilient to another drought in a quicker return interval. Lesser prairie‐chickens and other wildlife likely will benefit from habitat management that remains adaptive with continuous monitoring to ensure adequate habitat, including microhabitat, is available particularly during altered weather conditions. Based on results, specific adaptive measures that benefit wildlife on the Southern High Plains include destocking cattle during and immediately following severe drought, resting pastures between grazing events, and restocking at decreased levels following rainfall events in early spring. In addition, we suggest improving habitat quality and quantity to maintain stability and resiliency of species and entire systems through predicted changes in climate.

## CONFLICT OF INTEREST

None declared.

## AUTHOR CONTRIBUTION

All authors contributed to the manuscript including fund acquisition, data collection, data analysis, and writing/editing.
